# Identification of a Genotype VIId Newcastle Disease Virus Isolated from Sansui Sheldrake Ducks in Guizhou Province, China

**DOI:** 10.1128/genomeA.00161-15

**Published:** 2015-04-09

**Authors:** Zhiqiang Duan, Xinqin Ji, Houqiang Xu, Jiafu Zhao, Yong Ruan, Jiaqi Chen

**Affiliations:** a Key Laboratory of Animal Genetics, Breeding and Reproduction in The Plateau Mountainous Region, Ministry of Education, Guizhou University, Guiyang, China; b College of Animal Science, Guizhou University, Guiyang, China

## Abstract

In this study, we report the complete genome sequence of a novel Newcastle disease virus (NDV) strain, Sheldrake duck/China/Guizhou/SS1/2014, isolated from Sansui Sheldrake duck flocks in Guizhou Province, southwestern China. The genome of this isolate is 15,192 nucleotides in length, which belongs to NDV genotype VIId in class II. This discovery will help us further study the epidemiology characteristics and molecular pathogenesis of genotype VIId NDV in Sansui Sheldrake ducks.

## GENOME ANNOUNCEMENT

Newcastle disease (ND) is one of the virulent infectious diseases affecting the commercial poultry industry worldwide (1). The causative agent of ND is Newcastle disease virus (NDV), which is a member of genus *Avulavirus* within the family *Paramyxoviridae* (2). The genome of NDV is a negative-sense, single-stranded RNA of 15,186, 15,192, or 15,198 nucleotides in length (3, 4), which contains six genes encoding the nucleoprotein (NP), phosphoprotein (P), matrix protein (M), fusion protein (F), hemagglutinin-neuraminidase (HN), and large polymerase protein (L). NDV strains are divided into two distinct classes (I and II) based on genetic analysis. Almost all virulent NDV strains isolated from wild and domestic birds belong to class II, which can be further subdivided into 11 genotypes (5).

For a long time, waterfowl such as geese and ducks were considered to be the natural host of avirulent NDVs and were also thought to have been resistant to even the most virulent NDVs infecting chickens (6). However, outbreaks of velogenic NDVs in goose and duck flocks in southern and eastern China have aroused much concern since the late 1990s. Epidemiological studies have shown that genotype VII NDVs circulating predominantly in China are responsible for ND outbreaks in waterfowl flocks (7–9). In recent years, a small number of full genome sequences of different genotype NDVs isolated from domestic mallard ducks, including genotypes I and VII to IX (10–13), have been submitted to GenBank. However, complete genomic information for the genotype VII NDV isolated from Sansui Sheldrake ducks has not been reported.

In this study, an NDV strain that caused about 30% mortality was isolated from Sansui Sheldrake duck flocks in Guizhou Province, southwestern China. The isolate was named Sheldrake duck/China/Guizhou/SS1/2014 (SS1). The complete sequence of SS1 was amplified by 10 pairs of oligonucleotide primers and was subcloned into the TA cloning vector pCR 2.1 (Invitrogen, USA), followed by sequencing with an ABI3730 genome sequencer. Sequence analysis showed that the full genome sequence of SS1 is 15,192 nucleotides (nt) in length. Compared with the NDV vaccine strain LaSota (GenBank accession no. AF077761), there is a 6-nt (TCCCAC) insert in the 5′ noncoding region of the NP gene. The amino acid sequence identities of the F and HN proteins between SS1 and LaSota are 88.4% and 88.1%, respectively. From the sequence analysis, it has been found that the isolated NDV strain SS1 is significantly different than the vaccine strain, potentially leading to poor vaccination protection against the currently epidemic NDV.

The sequence at the F protein cleavage site is the major determinant of the NDV pathogenicity (14, 15). The F gene of the SS1 strain has the highest sequence homology (99.8%) to NDV strain JS-01-10-Dk (GenBank accession no. JQ013879, class II, genotype VII), and it has the same virulent F protein cleavage site sequence (^112^RRQKR↓F^117^), which is consistent with the detected biological characteristics (mean death time, 52 h; intracerebral pathogenicity index, 1.89; intravenous pathogenicity index, 2.80). The phylogenetic analysis by MEGA 5.2 of the F gene further suggests that the isolated SS1 strain belongs to NDV genotype VIId of class II.

### Nucleotide sequence accession number.

The complete genome sequence of Sheldrake duck/China/Guizhou/SS1/2014 has been deposited in GenBank under accession no. KP742770.
